# Neuroendocrine tumour masquerading as intussusception: A case report

**DOI:** 10.1016/j.ijscr.2020.10.091

**Published:** 2020-10-24

**Authors:** Lingwei Wong, Senthil Vasan Kanthasamy, Gunaseelan Durairaj, Ramesh R. Thangaratnam

**Affiliations:** a Hospital Serdang, Selangor, Malaysia; b University of Malaya, Kuala Lumpur, Malaysia; c Hospital Serdang, Jalan Puchong, 43000, Kajang, Selangor Darul Ehsan, Malaysia

**Keywords:** Neuroendocrine tumour, Adult intussusception, Case report

## Abstract

**Introduction:**

Intussusception usually occurs in the paediatric population. When it occurs in the adult population, it is normally caused by a malignant intraluminal pathology.

**Presentation of case:**

A 72-year-old female presented to us with right-sided abdominal pain for 3 weeks, associated with vomiting and diarrhoea. She had an appendectomy done 30 years ago and a recent myocardial infarction. Abdominal examination revealed a previous appendectomy scar and tenderness over the right lumbar region. Computed tomography showed ileocaecal intussusception. Right hemicolectomy with a double barrel stoma was performed as she was unstable intraoperatively. Histopathological examination of the tumour showed a well-differentiated neuroendocrine tumour. Subsequent PET scan showed no systemic disease and a reversal of the stoma was done. She remained disease free for a year.

**Discussion:**

Our patient had undergone a right hemicolectomy despite the high risk of mortality, as there is a high chance of malignancy. Double barrel stoma was done, as she was unstable intraoperatively. Fortunately, she recovered well and had her stoma reversed without any further recurrence of her disease.

**Conclusion:**

Adult patients who present with intussusception should be managed with resection, as there is a high possibility of a malignancy. Early resection should be planned to prevent further spread of the tumour.

## Highlights

• Intussusception commonly occurs in the paediatric population.
• Presentation in the adult population is likely malignant.
• It is important for an intussusception to be managed surgically in adults.
• Neuroendocrine tumour can present as intussusception in the adult population.
• Resection with lymph node clearance is needed for complete staging.

## 1 Introduction

Intussusception is rare in adults. It is a telescoping of a part of the intestine into itself. It is one of the common abdominal emergencies in the paediatric population, mainly between 6 and 36 months of age. Children with intussusception younger than one year old represent approximately 60% of intussusception cases children and about 80–90% of paediatric patients are younger than two years old [1].

When intussusception occurs, the mesentery is dragged into the bowel. This will cause congestion of the venous and lymphatic system, which would then cause bowel oedema. This will subsequently result in bowel ischaemia, perforation, peritonitis and eventually death. For intussusception to occur in adults, it is normally preceded by a pathological lead point, i.e. a polyp, duplication cyst, tumour, haematoma, vascular malformation, or a Meckel’s diverticulum [2].

Intermittent colicky abdominal pain, associated with nausea and vomiting, is the usual presentation, which can be similar to any other abdominal pathology [3]. Adult intussusception is associated with a tumour in about 63% of the cases [4]. Only a few cases are associated with ileocolonic intussusception secondary to carcinoid tumour [[5], [6], [7], [8]]. This case is unique as the patient had a previous appendectomy and presented with an adhesive obstruction with a recent myocardial infarction. This patient is managed in a tertiary centre with presence of general surgeons. This case has been reported in line with the Surgical Case Report (SCARE) guidelines [9].

## 2 Presentation of case

A 72-year-old female presented to us, a tertiary centre with right-sided abdominal pain for 3 weeks, associated with vomiting and diarrhoea. She had an appendectomy done 30 years ago and a recent myocardial infarction. She was admitted at that time and subsequently discharged well after an angioplasty was done. She has not complained of chest pain after that. She has no diabetes mellitus or hypertension. There is no family history of malignancy. She does not smoke nor drink alcohol. She is a homemaker and currently lives with her son.

On examination, the patient was alert, in mild discomfort and not cachexic looking. Her blood pressure was 130/80 mmHg, she had a regular pulse rate at 90 beats/min, and her temperature was 36 °C. She was not tachypneic and saturating well on room air. Her lungs were clear with good air entry. Examination of the abdomen revealed a previous appendectomy scar with no obvious hernia. Palpation of the abdomen revealed tenderness over the right lumbar region. Bowel sounds were normal. Her rectal exam and genitourinary exam were normal.

**Fig. 1:**
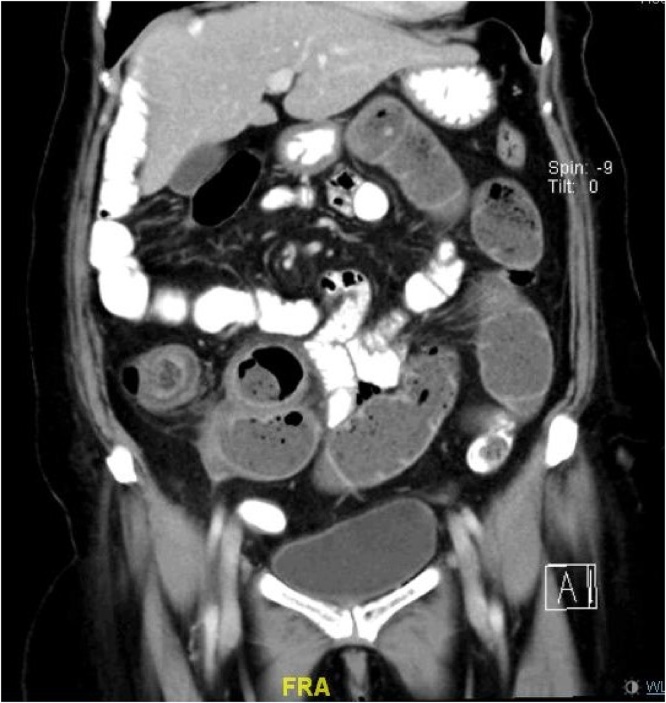
Intussusception seen on CT.

Her laboratory tests were unremarkable. Full blood count, electrolytes, liver function tests results, pancreatic enzymes and cardiac enzymes were all within normal limits. Ultrasound and computed tomography of the abdomen revealed ileocaecal intussusception (Fig. 1).

**Fig. 2:**
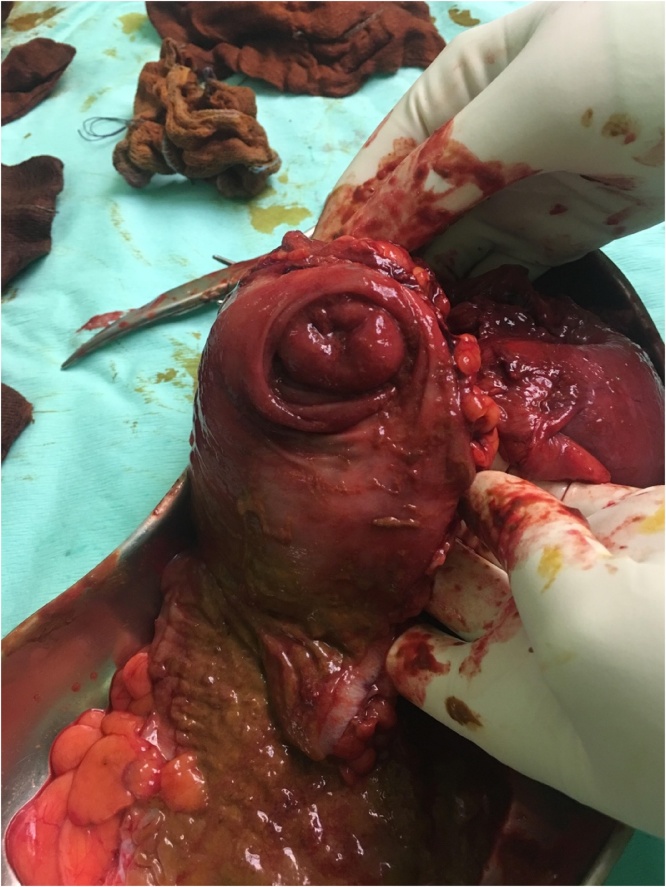
Intussusception with tumour within.

It was decided to proceed with surgery as the patient complained of persistent abdominal pain, despite the high risk of surgery due to the recent myocardial infarction. Laparotomy performed by the general surgeon on call showed ileocecal intussusception and right hemicolectomy was done, with double barrel stoma as the patient was haemodynamically unstable intraoperatively. The specimen was cut open and it showed a 1 cm tumour at the ileocaecal junction (Fig. 2).

**Fig. 3:**
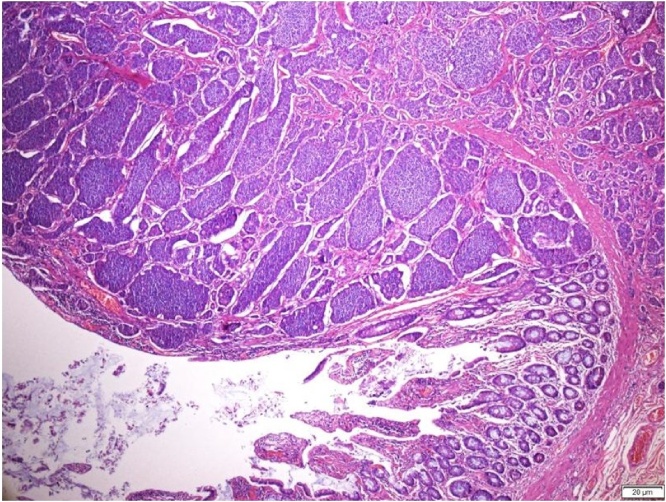
Mucosal-submucosal tumour with eroded overlying mucosa. The tumour cells form nests and trabeculae (H&E, ×40).

**Fig. 4:**
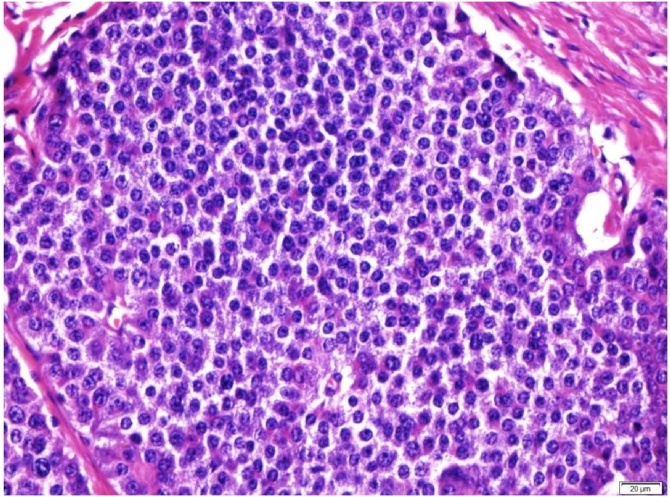
The tumour cells have round to oval nuclei with irregular chromatin clumps and small nucleoli. No mitotic activity seen (H&E, ×400).

**Fig. 5:**
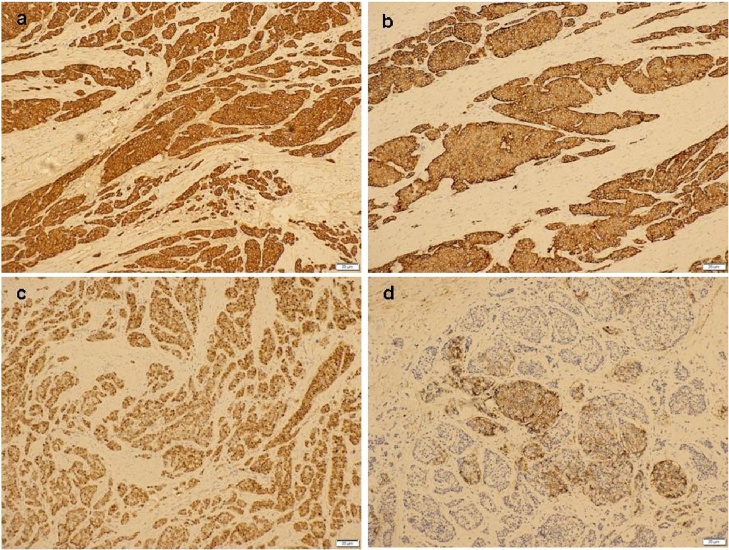
The tumour cells show diffuse immunoreactivity for Chromogranin A (a), Synaptophysin (b) and CDX2 (c) and focal immunoreactivity for CD56 (d).

Subsequent histopathological examination (HPE) showed tumour size measuring 2.3 cm × 1.8 cm × 2.0 cm at the ileocecal junction. It was a well-circumscribed submucosal tumour that bulged to the overlying mucosa without obvious mucosal ulceration. The tumour invaded into the muscularis propria and subserosa (Fig. 3). There was no obvious tumour perforation seen. Margins were clear of the tumour. There was no obvious mitosis identified (mitotic count <1/10 HPF) (Fig. 4). Lymphovascular and perineural invasions were present. The immunohistochemical studies showed the tumour cells were diffusely positive for CKAE1/AK3, chromogranin, synaptophysin and CDX2 and focally positive for CD56 (Fig. 5). The proliferative index Ki67 was <24. The impression was a neuroendocrine tumour, well differentiated Grade 1(carcinoid), mitosis: <2/10 HPF, Ki67: <2%. Lymph node involvement is 4/15. The pathological TNM staging was pT3pN1pMx, Dukes (Modified) C. She was referred to oncology and had a Ga-68 DOTANOC scan done that showed no systemic disease. No further adjuvant therapy was given. Reversal of the stoma was done about 9 months after her initial surgery, and she remained disease free during her follow up after 3 years. The patient was thankful to have survived the procedure despite her initial poor cardiac status.

## 3 Discussion

Neuroendocrine tumours in the small bowel are relatively rare, with the latest documented incidence in the United States of 3.56 per 100,000 population [10]. They can occur anywhere between the foregut, midgut and hindgut. There have been a few reported cases of neuroendocrine tumours that presented as intussusception [11].

Neuroendocrine tumours can present as non-functioning tumours, like in our patient, or present as functioning tumours, producing several peptides causing carcinoid syndrome. They can also be found incidentally during histopathological examination [12], just like in the case presented above. Symptoms of neuroendocrine tumours can range between intermittent abdominal pain, cutaneous flushing, diarrhoea and malabsorption, and cardiac manifestations leading to heart failure [13].

CT is normally done for the work up of these patient in the emergency setting and it would generally show the pathognomonic ‘target’ sign [14]. Carcinoid tumours are neuroendocrine tumour arising from enterochromaffin cells (Kulchitsky cells) and represent the most common tumours of the small intestine [15]. Neuroendocrine tumours of the gastrointestinal tract generally do not present with the classical symptoms of carcinoid syndrome unless they already metastasized. Symptoms associated with carcinoid are flushing, diarrhoea, abdominal colic, cardiac valve involvement on the right side and possible bronchial constriction.

Only a few cases of adult ileocaecal intussusception secondary to carcinoid tumour have been reported to date [[5], [6], [7], [8]]. A recent report by Zhang et al. [6], was on a patient who presented with haematochezia and abdominal pain. Their patient was worked up with a CT that confirmed intussusception and was planned for colonoscopy in an attempt to reduce the intussusception. The patient was not able to tolerate the procedure and laparotomy with right hemicolectomy was done with primary anastomosis. The HPE came back as neuroendocrine tumour but the patient has defaulted further follow up. Carrillo et al. [7] reported a large neuroendocrine tumour measuring 6 × 4 × 4 cm in size with liver metastasis. The patient underwent a right hemicolectomy with side to side ileotransverse anastomosis, liver biopsy and was referred to oncology for medical therapy; the patient and was reported to be well after 18 months. The two other cases were reported in Germany [5] and Portugal [8].

It appears that there is some form of variability in the treatment for adult patients with ileocecal intussusception but ultimately a right hemicolectomy should be done. In our patient, the risk of surgery is very high as she had a recent myocardial infarction. One might err on the side of doing a colonoscopic reduction for her to relieve the pain, in order to avoid general anaesthesia. She could also very well be having adhesion colic in view of her previous appendicectomy done, though that would be probably less likely in her. It was then decided that a resection is needed for her as the risk of malignancy is high. The decision was made to do a double barrel stoma for our patient as she would not have been able to survive an anastomotic leak.

Neuroendocrine tumours can be classified as well differentiated, low-grade (G1), well-differentiated, intermediate-grade (G2); poorly differentiated, high-grade (G3) [16]. It is staged according to the AJCC tumour (T), node (N), metastasis (M) staging system [16]. Prognostic markers for neuroendocrine tumours are Chromogranin A, mTOR and CDKN1B (p27). Elevated Chromogranin A is associated with poorer prognosis, mTOR over expression is associated with shorter overall survival [20] and mutations in the cyclin-dependent kinase inhibitor, CDKN1B (p27), is a poor prognostic factor in GI and pancreatic neuroendocrine tumour [17].

Resection is the primary treatment approach with regional lymph node clearance. Thorough examination of the entire bowel is also necessary, as multiple synchronous lesions may be present.

Surveillance should include complete patient history and physical examination with CT. Surveillance imaging of the chest may also be considered if clinically indicated in patients with primary GI tumours. Most patients with neuroendocrine tumours of the jejunum/ileum/colon, duodenum, rectum and thymus, and type 3 gastric neuroendocrine tumours with normal gastrin levels should be re-evaluated 3–12 months after resection, then every 12–24 months for up to 10 years. Chromogranin A may be used as a tumour marker, where elevated levels have been associated with recurrence [18].

In the evaluation of metastatic disease, serial measurement of 24-h urinary 5-HIAA can aid in evaluating for possible metastatic involvement or recurrence. This is because carcinoid tumours may secrete 5-hydroxyindole acetic acid (5-HIAA) and chromogranin A [18]. An octreotide scan is another modality to evaluate further metastatic disease during follow-up. PET/CT using radiolabelled somatostatin analogue gallium-68 has shown high sensitivity and specificity to detect patients with neuroendocrine tumour and metastasis [19].

Subsequent recurrence can be treated with somatostatin, which decreases the levels of serotonin secretion and decreasing the breakdown of serotonin. Symptom control using octreotide can be used for patients with metastatic disease.

## 4 Conclusion

Adult patients presenting with intussusception should be managed with resection as there is a high possibility of a malignancy. Early resection should be planned to prevent further spread of the tumour.

## Declaration of Competing Interest

The authors report no declarations of interest.

## Funding

None to declare.

## Ethical approval

This case report is exempt from ethical approval by our institution.

## Consent

Written informed consent was obtained from the patient for publication of this case report and accompanying images. A copy of the written consent is available for review by the Editor-in-Chief of this journal on request.

## Author’s contribution

Wong Lingwei: Conceptualization, Acquisition of data, Writing - original draft preparation.

Senthil Vasan Kanthasamy: Surgical therapy for this patient, Writing - Reviewing and Editing, Supervision.

Gunaseelan Durairaj: Surgical therapy for this patient, Writing - Reviewing and Editing, Supervision, Validation.

Ramesh R Thangaratnam: Surgical therapy for this patient, Writing - Reviewing and Editing, Supervision, Validation.

## Registration of research studies

N/A.

## Guarantor

Wong Lingwei.

## Provenance and peer review

Not commissioned, externally peer-reviewed.
